# Supplementary material for: Pyruvate Dehydrogenase Kinase 1 inhibition mediated oxidative phosphorylation enhancement in cartilage promotes osteoarthritis progression
Source: BMC Musculoskelet Disord. 2023 Jul 20;24:597. doi: 10.1186/s12891-023-06585-6 (PMC10357736; doi:10.1186/s12891-023-06585-6)
Supplement: Supplementary file 1 — Additional file 1. [file 12891_2023_6585_MOESM1_ESM.zip › BP.Gsea.1653623667859/GOBP_CELLULAR_RESPONSE_TO_TOXIC_SUBSTANCE.html]

Details for gene set GOBP\_CELLULAR\_RESPONSE\_TO\_TOXIC\_SUBSTANCE[GSEA]

|  || Dataset | 55457\_collapsed\_to\_symbols.cls.cls #OA\_versus\_normal.cls.cls #OA\_versus\_normal\_repos |
| Phenotype | cls.cls#OA\_versus\_normal\_repos |
| Upregulated in class | OA |
| GeneSet | GOBP\_CELLULAR\_RESPONSE\_TO\_TOXIC\_SUBSTANCE |
| Enrichment Score (ES) | 0.3369448 |
| Normalized Enrichment Score (NES) | 1.5365881 |
| Nominal p-value | 0.008174387 |
| FDR q-value | 0.3163709 |
| FWER p-Value | 1.0 |
Table: GSEA Results Summary

  

Fig 1: Enrichment plot: GOBP\_CELLULAR\_RESPONSE\_TO\_TOXIC\_SUBSTANCE      
 Profile of the Running ES Score & Positions of GeneSet Members on the Rank Ordered List

  

| SYMBOL | TITLE | RANK IN GENE LIST | RANK METRIC SCORE | RUNNING ES | CORE ENRICHMENT || 1 | PRDX4 | peroxiredoxin 4 [Source:HGNC Symbol;Acc:HGNC:17169] | 68 | 0.959 | 0.0256 | Yes |
| 2 | FBLN5 | fibulin 5 [Source:HGNC Symbol;Acc:HGNC:3602] | 69 | 0.958 | 0.0566 | Yes |
| 3 | PTGS1 | prostaglandin-endoperoxide synthase 1 [Source:HGNC Symbol;Acc:HGNC:9604] | 163 | 0.845 | 0.0766 | Yes |
| 4 | LTC4S | leukotriene C4 synthase [Source:HGNC Symbol;Acc:HGNC:6719] | 223 | 0.804 | 0.0980 | Yes |
| 5 | GSTK1 | glutathione S-transferase kappa 1 [Source:HGNC Symbol;Acc:HGNC:16906] | 490 | 0.662 | 0.0986 | Yes |
| 6 | ESD | esterase D [Source:HGNC Symbol;Acc:HGNC:3465] | 529 | 0.647 | 0.1165 | Yes |
| 7 | SOD3 | superoxide dismutase 3 [Source:HGNC Symbol;Acc:HGNC:11181] | 608 | 0.620 | 0.1304 | Yes |
| 8 | MGST3 | microsomal glutathione S-transferase 3 [Source:HGNC Symbol;Acc:HGNC:7064] | 610 | 0.620 | 0.1504 | Yes |
| 9 | SELENOT | selenoprotein T [Source:HGNC Symbol;Acc:HGNC:18136] | 639 | 0.609 | 0.1678 | Yes |
| 10 | AKR1A1 | aldo-keto reductase family 1 member A1 [Source:HGNC Symbol;Acc:HGNC:380] | 808 | 0.567 | 0.1730 | Yes |
| 11 | GSTZ1 | glutathione S-transferase zeta 1 [Source:HGNC Symbol;Acc:HGNC:4643] | 974 | 0.523 | 0.1770 | Yes |
| 12 | GSTM3 | glutathione S-transferase mu 3 [Source:HGNC Symbol;Acc:HGNC:4635] | 1025 | 0.512 | 0.1897 | Yes |
| 13 | GFER | "growth factor, augmenter of liver regeneration [Source:HGNC Symbol;Acc:HGNC:4236]" | 1075 | 0.500 | 0.2020 | Yes |
| 14 | GSTT1 | glutathione S-transferase theta 1 [Source:HGNC Symbol;Acc:HGNC:4641] | 1129 | 0.491 | 0.2137 | Yes |
| 15 | CLIC2 | chloride intracellular channel 2 [Source:HGNC Symbol;Acc:HGNC:2063] | 1214 | 0.473 | 0.2224 | Yes |
| 16 | GSTP1 | glutathione S-transferase pi 1 [Source:HGNC Symbol;Acc:HGNC:4638] | 1260 | 0.465 | 0.2339 | Yes |
| 17 | PARK7 | Parkinsonism associated deglycase [Source:HGNC Symbol;Acc:HGNC:16369] | 1406 | 0.442 | 0.2369 | Yes |
| 18 | SELENOF | selenoprotein F [Source:HGNC Symbol;Acc:HGNC:17705] | 1450 | 0.436 | 0.2476 | Yes |
| 19 | PRDX2 | peroxiredoxin 2 [Source:HGNC Symbol;Acc:HGNC:9353] | 1613 | 0.411 | 0.2482 | Yes |
| 20 | ALOX5AP | arachidonate 5-lipoxygenase activating protein [Source:HGNC Symbol;Acc:HGNC:436] | 1665 | 0.404 | 0.2572 | Yes |
| 21 | CAT | catalase [Source:HGNC Symbol;Acc:HGNC:1516] | 1731 | 0.394 | 0.2649 | Yes |
| 22 | ALDH1A1 | aldehyde dehydrogenase 1 family member A1 [Source:HGNC Symbol;Acc:HGNC:402] | 1737 | 0.393 | 0.2772 | Yes |
| 23 | GPX7 | glutathione peroxidase 7 [Source:HGNC Symbol;Acc:HGNC:4559] | 1919 | 0.370 | 0.2750 | Yes |
| 24 | SELENOW | selenoprotein W [Source:HGNC Symbol;Acc:HGNC:10752] | 1944 | 0.368 | 0.2850 | Yes |
| 25 | PRKN | parkin RBR E3 ubiquitin protein ligase [Source:HGNC Symbol;Acc:HGNC:8607] | 1972 | 0.364 | 0.2946 | Yes |
| 26 | NFE2L2 | "nuclear factor, erythroid 2 like 2 [Source:HGNC Symbol;Acc:HGNC:7782]" | 2034 | 0.357 | 0.3014 | Yes |
| 27 | TXNRD3 | thioredoxin reductase 3 [Source:HGNC Symbol;Acc:HGNC:20667] | 2049 | 0.356 | 0.3118 | Yes |
| 28 | PINK1 | PTEN induced kinase 1 [Source:HGNC Symbol;Acc:HGNC:14581] | 2097 | 0.350 | 0.3194 | Yes |
| 29 | TNF | tumor necrosis factor [Source:HGNC Symbol;Acc:HGNC:11892] | 2098 | 0.350 | 0.3307 | Yes |
| 30 | PRDX3 | peroxiredoxin 3 [Source:HGNC Symbol;Acc:HGNC:9354] | 2161 | 0.343 | 0.3369 | Yes |
| 31 | SOD1 | superoxide dismutase 1 [Source:HGNC Symbol;Acc:HGNC:11179] | 2413 | 0.313 | 0.3275 | No |
| 32 | MGST2 | microsomal glutathione S-transferase 2 [Source:HGNC Symbol;Acc:HGNC:7063] | 2583 | 0.292 | 0.3237 | No |
| 33 | ABCB6 | ATP binding cassette subfamily B member 6 (Langereis blood group) [Source:HGNC Symbol;Acc:HGNC:47] | 2717 | 0.278 | 0.3223 | No |
| 34 | CCS | copper chaperone for superoxide dismutase [Source:HGNC Symbol;Acc:HGNC:1613] | 2727 | 0.278 | 0.3306 | No |
| 35 | APOE | apolipoprotein E [Source:HGNC Symbol;Acc:HGNC:613] | 2776 | 0.272 | 0.3356 | No |
| 36 | PRDX1 | peroxiredoxin 1 [Source:HGNC Symbol;Acc:HGNC:9352] | 3113 | 0.239 | 0.3171 | No |
| 37 | APOM | apolipoprotein M [Source:HGNC Symbol;Acc:HGNC:13916] | 3415 | 0.210 | 0.3003 | No |
| 38 | GPX5 | glutathione peroxidase 5 [Source:HGNC Symbol;Acc:HGNC:4557] | 3558 | 0.198 | 0.2956 | No |
| 39 | NQO1 | NAD(P)H quinone dehydrogenase 1 [Source:HGNC Symbol;Acc:HGNC:2874] | 3635 | 0.191 | 0.2959 | No |
| 40 | OPRD1 | opioid receptor delta 1 [Source:HGNC Symbol;Acc:HGNC:8153] | 3663 | 0.189 | 0.2999 | No |
| 41 | RDH11 | retinol dehydrogenase 11 [Source:HGNC Symbol;Acc:HGNC:17964] | 4102 | 0.146 | 0.2704 | No |
| 42 | PTGES | prostaglandin E synthase [Source:HGNC Symbol;Acc:HGNC:9599] | 4399 | 0.121 | 0.2512 | No |
| 43 | GSR | glutathione-disulfide reductase [Source:HGNC Symbol;Acc:HGNC:4623] | 4597 | 0.105 | 0.2392 | No |
| 44 | ADH5 | "alcohol dehydrogenase 5 (class III), chi polypeptide [Source:HGNC Symbol;Acc:HGNC:253]" | 4720 | 0.094 | 0.2327 | No |
| 45 | ATP7A | ATPase copper transporting alpha [Source:HGNC Symbol;Acc:HGNC:869] | 4814 | 0.086 | 0.2282 | No |
| 46 | FANCC | FA complementation group C [Source:HGNC Symbol;Acc:HGNC:3584] | 5138 | 0.061 | 0.2049 | No |
| 47 | SESN1 | sestrin 1 [Source:HGNC Symbol;Acc:HGNC:21595] | 5204 | 0.056 | 0.2017 | No |
| 48 | DHFR | dihydrofolate reductase [Source:HGNC Symbol;Acc:HGNC:2861] | 5805 | 0.008 | 0.1551 | No |
| 49 | GPX2 | glutathione peroxidase 2 [Source:HGNC Symbol;Acc:HGNC:4554] | 5808 | 0.008 | 0.1552 | No |
| 50 | GSTM1 | glutathione S-transferase mu 1 [Source:HGNC Symbol;Acc:HGNC:4632] | 5848 | 0.005 | 0.1523 | No |
| 51 | MTARC2 | mitochondrial amidoxime reducing component 2 [Source:HGNC Symbol;Acc:HGNC:26064] | 6076 | -0.010 | 0.1349 | No |
| 52 | GSTO1 | glutathione S-transferase omega 1 [Source:HGNC Symbol;Acc:HGNC:13312] | 6275 | -0.025 | 0.1203 | No |
| 53 | TPO | thyroid peroxidase [Source:HGNC Symbol;Acc:HGNC:12015] | 6467 | -0.040 | 0.1067 | No |
| 54 | PRDX6 | peroxiredoxin 6 [Source:HGNC Symbol;Acc:HGNC:16753] | 6783 | -0.061 | 0.0840 | No |
| 55 | APOA4 | apolipoprotein A4 [Source:HGNC Symbol;Acc:HGNC:602] | 6817 | -0.064 | 0.0835 | No |
| 56 | TXNRD1 | thioredoxin reductase 1 [Source:HGNC Symbol;Acc:HGNC:12437] | 6820 | -0.064 | 0.0855 | No |
| 57 | GPX4 | glutathione peroxidase 4 [Source:HGNC Symbol;Acc:HGNC:4556] | 7115 | -0.089 | 0.0654 | No |
| 58 | HP | haptoglobin [Source:HGNC Symbol;Acc:HGNC:5141] | 7360 | -0.107 | 0.0498 | No |
| 59 | NXN | nucleoredoxin [Source:HGNC Symbol;Acc:HGNC:18008] | 7376 | -0.108 | 0.0521 | No |
| 60 | ABCG2 | ATP binding cassette subfamily G member 2 (Junior blood group) [Source:HGNC Symbol;Acc:HGNC:74] | 7473 | -0.116 | 0.0484 | No |
| 61 | MTARC1 | mitochondrial amidoxime reducing component 1 [Source:HGNC Symbol;Acc:HGNC:26189] | 7500 | -0.118 | 0.0502 | No |
| 62 | S100A9 | S100 calcium binding protein A9 [Source:HGNC Symbol;Acc:HGNC:10499] | 7509 | -0.119 | 0.0534 | No |
| 63 | GLRX | glutaredoxin [Source:HGNC Symbol;Acc:HGNC:4330] | 7554 | -0.123 | 0.0539 | No |
| 64 | FABP1 | fatty acid binding protein 1 [Source:HGNC Symbol;Acc:HGNC:3555] | 7604 | -0.127 | 0.0542 | No |
| 65 | PXDN | peroxidasin [Source:HGNC Symbol;Acc:HGNC:14966] | 7642 | -0.130 | 0.0555 | No |
| 66 | ALB | albumin [Source:HGNC Symbol;Acc:HGNC:399] | 7684 | -0.133 | 0.0566 | No |
| 67 | IPCEF1 | interaction protein for cytohesin exchange factors 1 [Source:HGNC Symbol;Acc:HGNC:21204] | 7800 | -0.141 | 0.0522 | No |
| 68 | ABTB2 | ankyrin repeat and BTB domain containing 2 [Source:HGNC Symbol;Acc:HGNC:23842] | 7916 | -0.149 | 0.0480 | No |
| 69 | CD36 | CD36 molecule [Source:HGNC Symbol;Acc:HGNC:1663] | 7985 | -0.154 | 0.0477 | No |
| 70 | LPO | lactoperoxidase [Source:HGNC Symbol;Acc:HGNC:6678] | 8298 | -0.178 | 0.0291 | No |
| 71 | AKR1B10 | aldo-keto reductase family 1 member B10 [Source:HGNC Symbol;Acc:HGNC:382] | 8420 | -0.187 | 0.0257 | No |
| 72 | HBB | hemoglobin subunit beta [Source:HGNC Symbol;Acc:HGNC:4827] | 8775 | -0.215 | 0.0049 | No |
| 73 | HBA1 | hemoglobin subunit alpha 1 [Source:HGNC Symbol;Acc:HGNC:4823] | 8789 | -0.216 | 0.0109 | No |
| 74 | GCH1 | GTP cyclohydrolase 1 [Source:HGNC Symbol;Acc:HGNC:4193] | 9465 | -0.271 | -0.0331 | No |
| 75 | TXN | thioredoxin [Source:HGNC Symbol;Acc:HGNC:12435] | 9487 | -0.273 | -0.0259 | No |
| 76 | BMP7 | bone morphogenetic protein 7 [Source:HGNC Symbol;Acc:HGNC:1074] | 9544 | -0.278 | -0.0213 | No |
| 77 | MPO | myeloperoxidase [Source:HGNC Symbol;Acc:HGNC:7218] | 9792 | -0.301 | -0.0309 | No |
| 78 | TXNRD2 | thioredoxin reductase 2 [Source:HGNC Symbol;Acc:HGNC:18155] | 9922 | -0.314 | -0.0308 | No |
| 79 | NOS3 | nitric oxide synthase 3 [Source:HGNC Symbol;Acc:HGNC:7876] | 9980 | -0.319 | -0.0250 | No |
| 80 | HBQ1 | hemoglobin subunit theta 1 [Source:HGNC Symbol;Acc:HGNC:4833] | 10473 | -0.370 | -0.0515 | No |
| 81 | EPX | eosinophil peroxidase [Source:HGNC Symbol;Acc:HGNC:3423] | 10492 | -0.372 | -0.0409 | No |
| 82 | HBZ | hemoglobin subunit zeta [Source:HGNC Symbol;Acc:HGNC:4835] | 10615 | -0.385 | -0.0380 | No |
| 83 | DUOX2 | dual oxidase 2 [Source:HGNC Symbol;Acc:HGNC:13273] | 10885 | -0.415 | -0.0456 | No |
| 84 | HBE1 | hemoglobin subunit epsilon 1 [Source:HGNC Symbol;Acc:HGNC:4830] | 11153 | -0.451 | -0.0519 | No |
| 85 | UBIAD1 | UbiA prenyltransferase domain containing 1 [Source:HGNC Symbol;Acc:HGNC:30791] | 11299 | -0.471 | -0.0480 | No |
| 86 | HBG1 | hemoglobin subunit gamma 1 [Source:HGNC Symbol;Acc:HGNC:4831] | 11391 | -0.485 | -0.0394 | No |
| 87 | GPX3 | glutathione peroxidase 3 [Source:HGNC Symbol;Acc:HGNC:4555] | 11407 | -0.488 | -0.0249 | No |
| 88 | GSTA1 | glutathione S-transferase alpha 1 [Source:HGNC Symbol;Acc:HGNC:4626] | 11608 | -0.517 | -0.0238 | No |
| 89 | DUOX1 | dual oxidase 1 [Source:HGNC Symbol;Acc:HGNC:3062] | 12336 | -0.687 | -0.0584 | No |
| 90 | HBD | hemoglobin subunit delta [Source:HGNC Symbol;Acc:HGNC:4829] | 12482 | -0.741 | -0.0458 | No |
| 91 | PIM1 | "Pim-1 proto-oncogene, serine/threonine kinase [Source:HGNC Symbol;Acc:HGNC:8986]" | 12500 | -0.748 | -0.0229 | No |
| 92 | PTGS2 | prostaglandin-endoperoxide synthase 2 [Source:HGNC Symbol;Acc:HGNC:9605] | 12641 | -0.820 | -0.0074 | No |
| 93 | SOD2 | superoxide dismutase 2 [Source:HGNC Symbol;Acc:HGNC:11180] | 12685 | -0.853 | 0.0168 | No |
Table: GSEA details [plain text format]

  

Fig 2: GOBP\_CELLULAR\_RESPONSE\_TO\_TOXIC\_SUBSTANCE      
 Blue-Pink O' Gram in the Space of the Analyzed GeneSet

  

Fig 3: GOBP\_CELLULAR\_RESPONSE\_TO\_TOXIC\_SUBSTANCE: Random ES distribution      
 Gene set null distribution of ES for **GOBP\_CELLULAR\_RESPONSE\_TO\_TOXIC\_SUBSTANCE**
